# Feature Papers in Population and Evolutionary Genetics and Genomics 2023: Unraveling Population Dynamics, Diversity, and Evolutionary Paths

**DOI:** 10.3390/genes15040446

**Published:** 2024-04-01

**Authors:** Maria-Anna Kyrgiafini, Zissis Mamuris

**Affiliations:** 1 Laboratory of Genetics, Comparative and Evolutionary Biology, Department of Biochemistry and Biotechnology, University of Thessaly, Viopolis, Mezourlo, 41500 Larissa, Greece; 2 Averofeio Agri-Food Technological Park of Thessaly, University of Thessaly, Gaiopolis, 41336 Larissa, Greece; 3 Institute of Animal Genetic Improvement, University Center for Research and Innovation PA.K.E.K. “IASON”, University of Thessaly, Viopolis, Mezourlo, 41500 Larissa, Greece

The dialogue between population genetics and evolutionary biology, which historically followed separate paths, has now developed into a complex and interdisciplinary field of study. This evolution reflects a broader trend in academia towards integrating research, bridging the gap between genetic microvariations within populations and the broader patterns of evolution seen in phylogenetic frameworks. Currently, the intertwined disciplines of population genetics, evolutionary biology, and genomics play a crucial role in unraveling the complexities of biological diversity and the underlying mechanisms that drive the origin and diversification of life. These disciplines meticulously analyze the genetic makeup of populations, shedding light on how genetic variation emerges, persists, and drives adaptation and speciation. Additionally, the emergence of genomics, driven by advanced sequencing methods and innovative technologies, has propelled these fields into a new era. This has allowed researchers to dissect complex genetic structures and evolutionary processes with unprecedented precision. Furthermore, the significance of population and evolutionary genetics extends beyond the academic realm, informing conservation strategies, agricultural practices, and medical research. This highlights the crucial role that these fields play in addressing global challenges related to biodiversity conservation, food security, and health, and it underscores the importance of genetic and evolutionary research in developing strategies for the sustainable management and conservation of biological resources.

This Special Issue, “Feature Papers in Population and Evolutionary Genetics and Genomics 2023”, brings together an engaging collection of eight cutting-edge articles that cover a wide range of crucial topics in evolutionary and population genetics. The collection comprises both original research and review articles, providing insights into studies on humans, animals, and plants. The Special Issue addresses complex subjects ranging from the population genomics of marine species and the genetic implications of environmental and societal changes on human populations to the genetic diversity within specific plant varieties. Each article contributes to a deeper understanding of the genetic and evolutionary dynamics at play, showcasing the interdisciplinary nature and breadth of contemporary research in these significant scientific fields.

More specifically, in the field of population genetics, Fu et al. (2023) [1] investigated the genetic diversity within the Han population of Luzhou by analyzing 24 Y-chromosome Short Tandem Repeat (Y-STR) markers. Engaging 910 participants, the study identified 893 distinct haplotypes, most of which are unique, thereby underscoring significant genetic polymorphism and a high capacity for discrimination. The results reveal close genetic affiliations between the Luzhou Han and adjacent Han populations, emphasizing the influences of geography, history, and economic development on genetic composition. This research is particularly noteworthy as it deepens the understanding of genetic relationships and population structures, offering crucial insights for forensic applications, especially in paternal lineage identification and broader population genetic studies. Furthermore, it is important to remember that the Han represents not only China’s largest ethnic group but also the most populous ethnic group globally. Another study in this field was that of Mikhailova et al. (2023) [2]. The study by Mikhailova et al. (2023) [2] explores whether prolonged socioeconomic hardships can have a genetic impact on human populations. Set against the backdrop of the Russian socioeconomic crisis in the 1990s, this research compares the genetic makeup of adolescents born during this challenging period with those born before and after. Using advanced PCR techniques, the researchers genotyped various genetic markers associated with stress resilience and stress-induced disorders. The study’s findings are intriguing and nuanced. While no significant differences were observed in the frequencies of many genetic markers tested, a notable exception was found in the *ESR1* gene. Adolescents born during the crisis had a notably higher frequency of the rs6557168 C allele compared to those from other periods, suggesting, according to the authors, a potential genetic adaptation or selection due to the prolonged stress caused by the socioeconomic crisis. Thus, this research opens the door to a deeper understanding of how significant environmental stressors can affect human genetics, potentially influencing future generations. It also provides essential insights into human resilience and adaptability in the face of hardship, emphasizing the need for further investigation in this area.

Regarding animal studies, Ramsay et al. (2023) [3] conducted a highly interesting study on habitat loss and forest fragmentation. Specifically, they examined how forest fragmentation affects the dispersal, connectivity, and genetic diversity of two rodent species in Madagascar: the endemic *Eliurus myoxinus* and the invasive *Rattus rattus*. The study was conducted in two landscapes in northwestern Madagascar, consisting of forest fragments and adjacent continuous forest patches. By analyzing genetic data (Restriction-site associated DNA sequencing, RADseq) from both species, the study revealed the differential impacts of forest fragmentation on endemic and invasive species. The lower genetic diversity and higher inbreeding observed in the endemic *E. myoxinus* suggest that this species may be more susceptible to the effects of fragmentation. In contrast, the invasive *R. rattus* appears to be more resilient, possibly due to its greater dispersal capabilities and adaptability to fragmented landscapes. Thus, the study underscores the varying impacts of fragmentation on endemic and invasive species, emphasizing the need for targeted conservation efforts that consider the unique ecological needs and vulnerabilities of endemic species. In another study, Fonseca et al. (2024) [4] utilized whole-genome sequencing to elucidate the genetic structure of the European sardine (*Sardina pilchardus*), a species of great commercial importance. By examining 108 individuals from 16 sampling areas spanning a distribution range of 5000 km, the study reveals a minimum of three distinct genetic clusters, indicating intricate population structures closely associated with geographical factors. These clusters include separate populations for the Azores and Madeira, the Iberian Peninsula, and the Mediterranean, with the Canary Islands showing genetic similarities with the latter. Importantly, the study reveals barriers to gene flow, such as the Almeria–Oran front, while also suggesting some degree of genetic admixture between regions. Identifying key genomic regions associated with genetic differentiation, particularly those linked to otolith formation, underscores the genetic basis behind population structure and potential evolutionary adaptations. These findings carry significant implications for fisheries management and conservation efforts, emphasizing the need for strategies tailored to specific regions that acknowledge the genetic distinctiveness and connectivity, or lack thereof, among populations. Thus, this study not only advances marine genomics but also emphasizes the critical role of comprehensive genomic data in developing effective conservation strategies that promote the sustainable utilization of marine resources.

This Special Issue also encompasses studies dedicated to plant species. The study conducted by He et al. (2023) [5] utilized oligonucleotide fluorescence in situ hybridization (Oligo-FISH), an advanced cytogenetic technique, to investigate the chromosomal characteristics and genetic relationships of five *Zanthoxylum armatum* varieties/cultivars, a plant known for its medicinal and culinary properties. The research revealed significant karyotypic variations among the *Z. armatum* varieties/cultivars, underscoring the species’ genetic diversity. Additionally, the study pinpointed unique chromosomal markers and clarified the phylogenetic relationships within *Z. armatum*, providing further insight into its genetic diversity and evolutionary paths. These findings have important implications for conservation genetics, and simultaneously, the methodology employed in this study proves valuable in distinguishing between various *Z. armatum* varieties and cultivars. Thus, this research not only expands our knowledge of the genetic landscape of *Z. armatum* but also underscores the critical role of genetic and chromosomal analysis in the field of plant biodiversity, with potential applications for conservation strategies, breeding programs, and the field of pharmacognosy.

A quite different but extremely interesting study brings to the table the subject of genotype imputation, a widely used approach to enrich genetic datasets. More specifically, the study conducted by Dekeyser, Génin, and Herzig (2023) [6] examines the impact of reference panel diversity on genotype imputation accuracy using a new method that involves inserting synthetic genetic variants to track haplotype contributions across different genomic regions. The findings show that while diversity in reference panels generally improves imputation performance, it can sometimes result in the imputation of incorrect genotypes. To address this issue, the researchers propose a technique that leverages the benefits of diversity while mitigating its adverse effects, providing a more nuanced understanding of the role of diversity role in reference panel effectiveness. This approach represents a significant advancement in optimizing reference panel selection, with potential implications for enhancing genetic analyses in research and personalized medicine. The study’s insights into balancing diversity and accuracy in reference panels could inform the future development of imputation software, making it a valuable contribution to the field of genomics.

The study by Xiang et al. (2023) [7] also provides a significant advancement in our understanding of genetic mobility and diversity as it presents new information about the distinct evolutionary profile of the DD37E/Mosquito (MS) family within the *Tc1/Mariner* transposable elements. By employing sophisticated bioinformatics analyses, the researchers have meticulously delineated the MS elements as forming a unique monophyletic clade, starkly divergent from the previously recognized DD37E/L18 and DD37E/TRT groups. This revelation is particularly noteworthy as it underscores the MS family’s exclusive presence in invertebrates, predominantly within the Arthropoda phylum, thereby enriching our comprehension of the taxonomic distribution of transposable elements. The structural and functional characterization of MS elements provides invaluable insights into the mechanics of transposition and the evolutionary strategies employed by these genetic entities. Furthermore, another interesting discovery is the occurrence of horizontal transfer events across dipteran lineages. This highlights the dynamic nature of MS transposons and their potential role in shaping genomic architectures across different biological lineages. Overall, this study contributes to our understanding of the evolutionary history of the *Tc1*/*Mariner* superfamily. It also opens new avenues for the study of active transposons. Such insights are pivotal for the broader scientific community, offering a deeper understanding of genetic evolution mechanisms and potentially informing future research in genetics, biodiversity conservation, and the development of innovative biotechnological applications.

Finally, an extremely interesting review was also included in this Special Issue. The article by Török (2023) [8] explores the complex population history of the Carpathian Basin, shedding light on the genetic and linguistic heritage that shapes modern Hungarians. Based on an in-depth genomic analysis of ancient populations, including the Huns, Avars, and conquering Hungarians, the research reveals a multifaceted interplay of migrations and admixtures. Key findings demonstrate that modern Hungarians have a predominantly European gene pool despite historical Asian influences. Furthermore, a significant discovery is the presence of the “Conqueror Asia Core” genetic component in the elite ancient Hungarian population, connecting them to a proto-Ugric group from northern Kazakhstan. This finding aligns with linguistic data suggesting a shared Ugric proto-language, highlighting the intricate relationship between genetics and linguistics in the region’s history. Thus, the study’s impact extends beyond historical genetics, providing insights into broader processes such as human migration, cultural integration, and the formation of linguistic identities. Importantly, it also emphasizes the necessity of a multidisciplinary approach that synthesizes genetic, linguistic, and archaeological data to unravel the complex tapestry of human history in the Carpathian Basin and beyond.

In conclusion, the Special Issue “Feature Papers in Population and Evolutionary Genetics and Genomics 2023” presents a rich tapestry of studies that advance our understanding of genetic diversity, evolutionary dynamics, and population structures across various species and contexts. Collectively, these papers expand our knowledge and provide a foundation for future research. They emphasize the urgent need to further explore genetic diversity and connectivity, the effects of environmental changes on genetic structures, and the need to improve genetic analysis tools. This Special Issue not only guides future investigations but also underscores the importance of a multidisciplinary approach in unraveling the complexities of evolution and genetics, paving the way for advancements in conservation strategies, medical genetics, and our understanding of human history.
